# Pain, disease severity and associations with individual quality of life in patients with motor neuron diseases

**DOI:** 10.1186/s12904-021-00848-6

**Published:** 2021-10-12

**Authors:** Ylva Åkerblom, Lena Zetterberg, Birgitta Jakobsson Larsson, Dag Nyholm, Ingela Nygren, Pernilla Åsenlöf

**Affiliations:** 1 Department of Neuroscience, Physiotherapy, Uppsala University, Box 593 BMC, 751 24 Uppsala, Sweden; 2 Department of Public Health and Caring Sciences, Uppsala University, Box 564, 754 22 Uppsala, Sweden; 3 Department of Neuroscience, Neurology, Uppsala University, 75185 Uppsala, Sweden

**Keywords:** Amyotrophic lateral sclerosis (ALS), Palliative diseases, Individual quality of life, Pain, Disease severity

## Abstract

**Background:**

Up to 85% of people with motor neuron disease (MND) report pain, but whether pain has negative impact on quality of life is unclear. The aim was to study associations between pain, disease severity and individual quality of life (IQOL) in patients with MND.

**Methods:**

In this cross sectional study, 61 patients were recruited from four multidisciplinary teams in Sweden, whereof 55 responded to the pain measure (The Brief Pain Inventory – Short form) and were included in the main analyses. Disease severity was measured with the Amyotrophic Lateral Sclerosis Functional Rating Scale - Revised Version, and individual quality of life was measured with a study-specific version of the Schedule for the Evaluation of Individual Quality of Life - Direct Weighting.

**Results:**

Forty-one (74%) of the participants who answered BPI-SF (*n* = 55) reported pain. Thirty-nine (71%) of those reported pain during the past 24 h. The severity of pain was on average moderate, with eight participants (14%) reporting severe pain (PSI ≥ 7).

Satisfaction with IQOL for the entire sample was good (scale 1-7, where 1 equals poor quality of life): median 5, interquartile range (IQR) 2.75 and there was no difference in satisfaction with IQOL between those reporting pain/not reporting pain (median 5, IQR 2/median 5, IQR 3.5, Mann-Whitney U = 249, *p* = 0.452). There was neither any correlation between pain severity and satisfaction with IQOL, nor between disease severity and satisfaction with IQOL.

**Conclusions:**

The results add to the hypothesis that associations between non-motor symptoms such as pain prevalence and pain severity and IQOL in MND are weak. Pain prevalence was high and the results pointed to that some participants experienced high pain severity, which indicate that pain assessments and pain treatments tailored to the specific needs of the MND population should be developed and scientifically evaluated.

## Background

Motor neuron disease (MND) is due to degeneration of motor neurons in the spinal cord, brainstem and motor cortex. Symptom representation varies but commonly includes muscle wasting, weakness, spasticity, cramps, dyspnea, dysarthria, and dysphagia [1]. Besides motor symptoms, anxiety, depression, limited cognitive function, and sensory disturbances e.g. pain, may also be present [1, 2]. Amyotrophic lateral sclerosis (ALS) is the most common MND diagnosis with a survival from symptom onset to usually 3-5 years [1]. The incidence for ALS in Europe is estimated to 2.2/100000 persons a year [3] and the prevalence is around 6/100000 [4]. Primary Lateral Sclerosis (PLS) is another MND which affects the upper motor neurons with spasticity of the limbs as primary symptom. Persons with PLS have more benign prognosis than those with ALS [5]. There are also other rare subtypes of MND, only affecting lower motor neurons, for instance progressive spinal muscular atrophy (PSMA). Motor neuron disease is incurable, which means that palliative care often starts when the patient has received the diagnosis [6].

Pain is common in MND and affects up to 85% of the MND population [7–11]. Pain seems to be present in all stages of the disease [12, 13]. The severity of pain is reported mild to moderate [2, 7–10] and appears to affect personal daily functioning [7, 8, 10]. Even though the World Health Organization’s (WHO) definition of palliative care underlines the importance of measures for pain relief [14], there is no evidence based pharmacological treatment for pain in ALS [15]. Non-pharmacological intervention studies are few and do not support one treatment over the other [16–18], and treatment recommendations are based on guidelines for non-cancer chronic pain combined with clinical experience of treating patients with ALS and pain [19].

Health related quality of life (HRQOL) concerns aspects of physical, mental and social functioning on health [20] and thereby includes how disease affects disability and every day function [21, 22]. Health related quality of life in people with MND worsen during disease progression [23, 24]. In degenerative neuromuscular diseases, an awareness regarding the potential inappropriateness of asking people about their HRQOL has emerged. Instead, individual quality of life (IQOL) is emphasized as more relevant, since it encompasses the personal and unique meaning of what constitutes QOL [25]. More precisely, IQOL focuses on a person’s own perspectives of QOL, based on areas in life considered to be important at the present time [26]. Important areas of IQOL in people with MND are family, social activities and psychosocial functioning [27–31]. Despite the severe and fatal prognosis, people with MND consistently rate their IQOL as high during the course of the disease [30–32]. Hence, the physical deterioration due to disease progression does not seem to affect IQOL in people with MND [29, 30, 32, 33]. A shift in values regarding what is important in life when suffering from an incurable disease, might be the explanation to a maintained high IQOL throughout disease progression [25].

Research on associations between pain and QOL in people with MND show ambiguous results. Ganzini et al. reported that pain correlated to suffering, but not to QOL [34]. Another study showed that pain did not correlate to QOL [2], while a third study found that higher pain intensity predicted worse QOL until controlling for depression [11]. To our knowledge there are no studies on associations between pain and IQOL in people with MND. Such knowledge is regarded as key in palliative care for people with MND, which motivated the present study.

## Methods

The aim was to study associations between pain prevalence, pain severity, disease severity and IQOL in patients with MND.

### Design

The design was correlational using interview and survey data from the first point of measurement in a prospective study with all in all five measurement occasions. Non-parametrical statistical analyses were used for the main analyses, whereas data on what constitute IQOL were analyzed qualitatively.

### Settings and participants

Patients were recruited from four multidisciplinary MND teams in Sweden from September 2015 to September 2016. The sample size was determined by number of eligible participants enrolled in the four teams during this time period. In accordance with ordinary routines, the patients were scheduled for meetings with either the whole or a part of the team about every 10 weeks. The teams were multidisciplinary including a neurologist, a nurse, a physiotherapist, an occupational therapist, a social worker, a speech therapist and a dietician.

Patients, who had a scheduled visit to any of the MND teams during the recruitment period, had an MND diagnosis [4] and were over 18 years old were asked to participate in the study. The patients could be in different stages of the disease. Patients with Kennedy’s disease were excluded mainly due to the sensory impact of the disease that might affect their perception of pain in a different way compared to the other motor neuron diseases included [35]. Patients with impact on cognitive function, i.e. difficulties in understanding the instructions for the study; patients with difficulties in understanding or expressing themselves in Swedish and patients with another neurological disease affecting the symptoms of the MND, were also excluded.

### Measures

*Individual quality of life* was assessed with a study-specific version of the Schedule for the Evaluation of Individual Quality of Life Direct Weighting (study-specific SEIQoL-DW) [26, 36]. The instrument is based on a semi-structured interview and started with an open question; “If you think about your whole life situation as it is right now, what are the most important areas, both good and bad, that are vital for your quality of life?” [26]. Thereafter, participants were asked to: 1. identify the most important areas of quality of life, and describe the meaning of each area, 2. select five of the areas, which currently are the most important, 3. rate how satisfied you are with respect to each of the five areas [26]. The original version includes a weighting procedure that was omitted in this study-specific version, since it does not seem to impact the total index of IQOL [37]. Level of satisfaction was rated on seven-point categorical scales, with the following response format: 1= “as bad as could possibly be”, 2 = “very bad”, 3 = “bad”, 4 = “fairly good”, 5 = “good”, 6 = “very good” and 7 = “as good as could possibly be” [36]. The study-specific version used categorical scales instead of the original visual analogue scales to decrease efforts and motor skills required from the respondent. The latter require that a line is drawn corresponding to level of satisfaction for each scale, which can be demanding for participants with decreased motor function.

An IQOL score was calculated for each participant using the median of the ratings for the five areas. The IQOL index score for the total sample was then calculated using the median of the IQOL scores [37]. The SEIQoL-DW has been presented as a valid measure for measuring IQOL in different diagnoses including ALS [38, 39].

*Pain* was measured with the Short Form of Brief Pain Inventory (BPI-SF) [40]. The BPI-SF measures the presence of pain, pain severity, body regions affected, treatments for pain and pain interference in different activities [40]. In the present study, three subscales were used; presence of pain during the past 24 h, body regions affected, and pain severity. Presence of pain during the past 24 h was indicated with “yes/no”. Then, the participant was asked to mark painful areas on the body with help of a mannequin. Severity of pain was rated on four 11-point numeric rating scales (NRS) for worst, least, and average pain intensity during the past 24 h, and for current pain. The anchors were labelled: 0 = “no pain” and 10 = “worst imaginable pain” [41]. The Pain Severity Index (PSI) was established by the average ratings of BPI-SF of worst, average, and current pain. An average of 0-3 is considered no or mild, 4-6 as moderate and 7-10 as severe pain [42]. The instrument is considered to be valid for several painful conditions [43–45] and has been widely used to evaluate pain in neuromuscular disorders including ALS [7–10, 46]. The internal reliability is high with Chronbach’s α 0.84 - 0.93 in participants with non-cancer pain, systemic lupus erythematosus and musculoskeletal pain [43–45].

*Disease severity* was assessed with The Amyotrophic Lateral Sclerosis Functional Rating Scale - Revised Version (ALSFRS-R) [47]. It includes four subscales measuring bulbar, fine motor, gross motor, and respiratory function. Each subscale includes three items ranging from 0 (no function) to 4 (full function) making a total score of 12 for each of the subscales. Lower scores indicate a higher level of dysfunction [47]. The internal consistency reliability is high, Cronbach’s α 0.73 [47]. The construct validity of the total score correlates with HRQOL measured with the Sickness Impact Profile, r_s_ = −.72 and with pulmonary function (forced vital capacity %) r_s_ = .41 [47].

*Demographic data* included screening for neuropathic pain [48, 49], sex, age, family situation, education, occupational status, MND-diagnosis, time since first symptom of the MND, pharmacotherapy and chronic pain experienced before onset of the MND. The data were collected from the participants at the clinical visit except from data on pharmacotherapy, which were derived from the participants’ medical records.

### Procedures

The study was approved by the Regional Ethics Committee in Uppsala, Sweden (approval No. 2015/293). Four MND teams in Sweden were asked to participate in the study and all accepted to participate. Before the start of the study, the first author (YÅ) informed the data collectors from the different teams about the study procedures and measures and provided them with written information. The number of data collectors in the teams varied from two to six. Eight of the total 15 data collectors were physiotherapists, three were nurses, one was occupational therapist and three were physicians.

Participants were recruited by either the coordinator of the multidisciplinary MND team or the data collector, who sent information letters to eligible participants some weeks before their scheduled clinical visit to the MND team. At the visit, the patients received oral information about the study. The patients confirmed participation by signing an informed consent form. Data were then collected during the clinical visit. In order for the data collection to be at a reasonable length, the BPI-SF [40] was completed at home either on paper or by computer. For those who did not immediately send in their forms, written reminders were sent at the most three times.

### Data management and analysis

Data from item 1 and 2 in SEIQOL-DW were categorised using a qualitative content analysis. Two of the authors (YÅ and BJL) initiated the analytic process by grouping areas based on what the participant brought up and how they described them. In the next step, the authors PÅ and LZ joined the process and contributed with further analyses and re-grouped areas when new consensus was reached. The analysts had different experiences and competencies, where two of the authors (YÅ, BLJ) had extensive experience of clinical care and physiotherapy treatment for patients with MND and one of physiotherapy treatment of patients with neurological diseases (LZ), and one (PÅ) of physiotherapy and interdisciplinary treatment of patients with chronic pain. One of the authors was a registered nurse (BLJ) the others were registered physiotherapists (YÅ, LZ, PÅ). A language editor was involved in the translation process of patients ‘quotations to secure that their content was kept although translated from Swedish to English.

Non-parametric statistics were used due to data level (ordinal) and data not being normally distributed. Descriptive statistics were used to specify a) the IQOL index (median) i.e. the IQOL on a group level, b) the number of participants with and without pain respectively who nominated each area c) how satisfied the participants were with each nominated area (median), d) participants’ pain severity related to each area (mean) e) participants’ bulbar, fine motor, gross motor, and respiratory function related to each area. The between-group difference in IQOL scores for participants with and without pain was analyzed with Mann-Whitney U-test. Univariate correlations were calculated between the IQOL score for each participant and pain severity (BPI-SF) and between the IQOL score for each participant and disease severity (ALSFRS-R) using the Spearman’s rho. For the latter, calculations were done separately for the four subscales of bulbar function, fine motor function, gross motor function, and respiratory function as recommended by Bakker et al. [50]. A significance level of *p* ≤ 0.05 was set. All statistical analyses were performed with the software version SPSS IBM statistics 24.

## Results

**Fig. 1:**
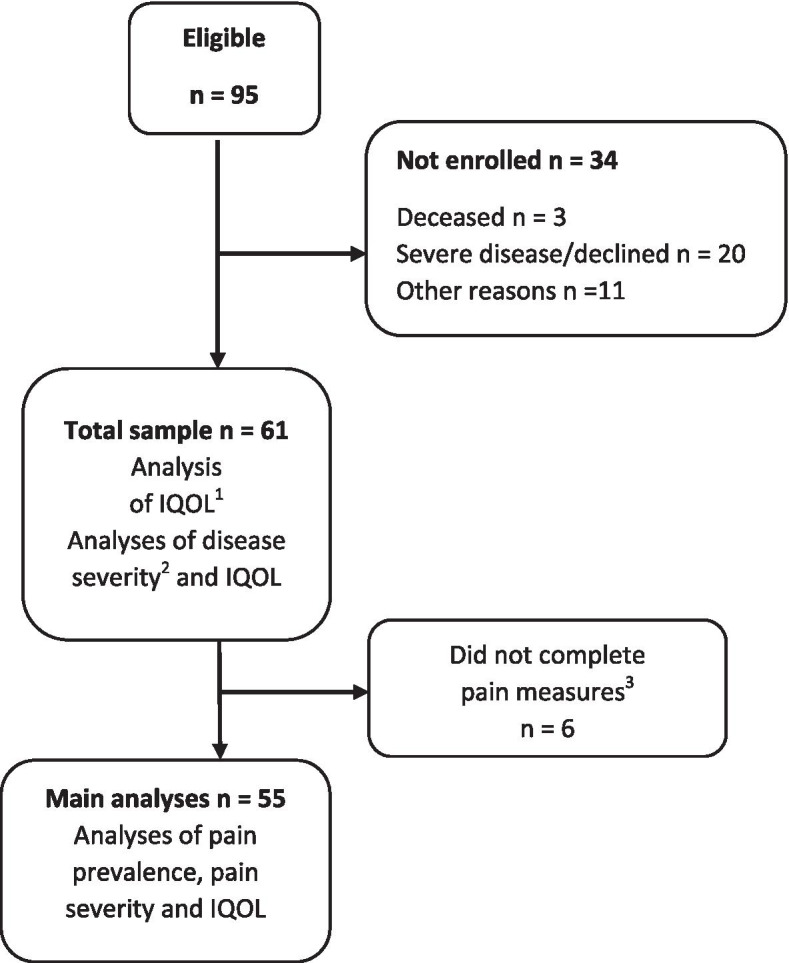
Patient flow. ^1^ Individual Quality of Life measured with a modified version of the Schedule for the Evaluation of Individual Quality of Life - Direct Weighting. ^2^ Measured with the Amyotrophic Lateral Sclerosis Functional Rating Scale-Revised version. ^3^ Pain prevalence and pain severity measured by the Brief Pain Inventory - Short Form

There were in total 154 patients enrolled in the four multidisciplinary teams during the recruitment period. Ninety-five patients met the criteria for inclusion, whereof 61 consented to participate in the study. Figure 1 shows participant flow and number of participants included in the main analyses. No imputations were made for missing data (see Fig. 1).

### Participants’ characteristics

Men were almost twice as many as the women (men *n* = 39, women *n* = 22). Most of the participants were retired or had fulltime sick leave. The majority (88.5%) had either ALS or an MND with only lower motor neuron signs and symptoms and the rest had PLS. Gross and fine motor functions were more commonly affected compared to the bulbar and respiratory functions. Forty-one (74%) of the participants who answered BPI-SF (*n* = 55) reported pain. Thirty-nine (71%) of those reported pain during the past 24 h. The severity of pain was classified as moderate (PSI mean = 3.8, SD = 2,4), with eight participants (14%) reporting severe pain (PSI ≥ 7). Participants’ characteristics are reported in Table 1.

**Table 1:** Patient characteristics

Patients characteristics, n	All patients, 61
**Gender**, male/female n (%)	39/22 (64/36)
**Age**, all patients, m (SD)	61.9 (12.3)
**Family situation**, n (%)	
Married/cohabitant	35 (57)
Partner and children	11 (18)
Single parent	3 (5)
Single	12 (20)
**Education**, n (%)	
Elementary school	15 (25)
High school	24 (39)
University	22 (36)
**Occupational status**, n, (%)	
Working full-time	6 (10)
Working part-time	5 (8)
Sickness benefit fulltime	17 (28)
Retired	31 (51)
Unemployed	1 (2)
Other	1 (2)
**Diagnosis**, n (%)	
ALS^a^	31 (51)
MND^b^	23 (38)
PLS^c^	7 (12)
**Time since first symptom of disease**, m year (std)	5,8 (7.2)
**Pain**, BPI-SF^d^, *n* = 55	
Pain, *n* = yes (%)	41 (74)
Pain during past 24 h, *n* = yes (%)	39 (71)
Worst level of pain, md (IQR)	5.0 (4.5)
PSI, *n* = 41, m (std)	3,8 (2,4)
**Neuropathic pain**, n (%), DN4^e^	12 (20)
**Chronic pain before onset of MND**, n (%)	22 (36)
**Disease severity**, ALSFRS-R^f^, md (Q_1_-Q_3_)	
The bulbar function	10 (7,5-12)
The fine motor function	8 (4-10)
The gross motor function	7 (5-9)
The respiratory function	12 (10-12)
**Drugs**^g^, n (%)	
Analgetics	6 (10)
Antidepressants	17 (28)
Antiepileptics	2 (3)
Anxiolytics	9 (15)
Hypnotics	16 (26)
NSAID	8 (13)
Opioids	7 (12)
Spasmolytics	6 (10)
Triptans	2 (3)
Riluzole	55 (90)
Nothing	2 (3)

^a^*ALS* Amyotrohic Lateral Sclerosis with both upper and lower motor neuron signs and symptoms but not further classified into the specific El Escorial categories

^b^*MND* Motor Neuron Disease with lower motor neuron signs and symptoms

^c^*PLS* Primary Lateral Sclerosis with only upper motor neuron signs and symptoms

^d^*BPI-SF* Brief Pain Inventory Short Form is a self-rating questionnaire about pain. Pain intensity for worst, least, average and current pain is graded from 0 (no pain) to 10 (pain as bad that you can imagine), *PSI* Pain Severity Index is the average score of worst, average and pain perceived at the time of the interview. No/mild PSI are considered between score 0-3, moderate PSI between scores 4-6 and severe PSI 7-10

^e^*DN4* Doleur Neuropathique 4 questions – Swedish version is a screening measurement of neuropathic pain

^f^*ALSFRS-R* Amyotrophic Lateral Sclerosis Functional Rating Scale Revised version is a 12-item scale of disease severity of Amyotrophic lateral sclerosis. ALSFRS-R assesses the level of function in the four domains of bulbar function, fine motor function, ross motor function and respiratory function. Each item is rated from 0 (worst) to 4 (best), corresponding to a total score of maximum 48

^g^Drugs, Riluzole, improves survival in people with ALS in about 3-6 months and might have a beneficial effect on neuropathic pain

### What constitutes quality of life for people with MND

The participants nominated altogether 19 areas of importance for their IQOL. The areas are presented in Table 2 together with examples of participants’ quotations illustrating the meaning of each area. The areas that most participants described as important for IQOL were “*Social relations” n* = 35/12 (participants with pain/without pain)*,* followed by “*Activities for amusement and relaxations” n* = 23/4 and “*Being in the outdoor environment” n* = 12/7 (Table 3). Five of the areas were only mentioned by the participants who reported pain. These areas were: “*A safe and comfortable home environment”, “A pet”, “Hope for the future”, “Having a philosophy of life”,* and *“Being alone”*.

**Table 2:** Important areas of quality of life as described by the participants (*n* = 61). Data collected with a modified version of the Schedule for the Evaluation of Individual Quality of Life - Direct Weighting

**Areas**	**Description of area content**	**Examples of participants’ quotations.**
Social relations *n* = 47	Social contacts, relations and interactions that make one feel happy, comfortable and taken care of, also including the possibility to care for other people.	“To be with my friends, the social part, to laugh and hang around” (37)^a^
	The importance of collegial fellowship*.*	“To take and give love with my children” (7)
		“The family and my friends - often contact and are close” (50)
		“My marriage - the thoughtfulness and full of fun” (58)
		“The work - the community (28)
Activities for amusement and relaxation *n* = 27	Engagement in hobbies e.g. relaxing activities, entertainments at the theatre or sport events, watching TV, reading books, Internet browsing, listening to music, daydreaming, singing, taking photographs.	“ Cross-word to activate the brain” (56)
		“Theater visit - the entertainment” (9)
		“Listen to music” (18)
		“Take pictures to get the perfect picture” (36)
		“Watch sport on TV” (39)
		“Listen to a book” (48)
		“Needlework” (57)
Being in the outdoor environment *n* = 19	Activities in the outdoor environment and nature e.g. fishing, berry picking.	“Walk in the nature with the dog, love the nature” (47)
Being independent *n* = 13	Being able to decide things by yourself and transport myself.	“To feel free and make own decisions” (7)
		“The car - a sense of freedom to transport oneself without help” (16)
Being able to work *n* = 11	Having an occupation with daily routines. A sense of coherence; feeling important and capable at work*.* Better economy.	“The work - do something meaningful” (15)
		“The work - better economy” (6)
		“Manage to work, to have something to do” (59)
		“The work - to be someone and discuss (41)
Being physically active *n* = 11	The ability to be physically active	“To be able to perform the daily exercise” (40)
Access to support and aids *n* = 10	Having access to support from health-care, including personal assistance, psychological support and aids	“Aids - BiPAP, cough assist, indoor wheelchair facilitates physically and psychologically” (33)
		“That people around me make it possible for me to feel free” (7)
Get along with daily inconveniences *n* = 9	Being happy of things that work in the daily life and appreciation of being spared from e.g. a cold, pain or other discomforts.	“To feel pretty good, meaning not too much nausea or too much pain” (34)
		“To be able to eat without too much problem with mucus” (22)
		“To not have a cold” (23)
		“Be able to talk” (46)
Being in good health *n* = 7	Feeling as good as possible	“To feel well inside and have a good mood” (1)
A safe and comfortable home environment *n* = 6	Feeling comfortable and secure at home, both indoors and outdoors, for both oneself and others.	“The home – with its safety and stability” (39)
		“That everyone feel comfort with our home environment” (27)
		“A good house close the nature” (61)
		A safe home with food for the day, where the wife is cooking” (26)
The cottage *n* = 6	The sense of peace and satisfaction from being at one’s cottage.	“My cottage – a sense of happiness and freedom” (41)
Enjoying good food and drinks *n* = 5	To enjoy and cook good food and drinks including eating at restaurants.	“Go to restaurant and eat good food (50)
		“Cook good tasted food” (36)
		“The food and wine” (48)
Being able to travel *n* = 5	The joy of making a journey.	“Travel and experience other environments and people” (6)
Domestic care and family responsibilities *n* = 3	Being able to take care of household chores and the family.	“Manage the household which works today, vacuum-clean, dishwasher and washing machine” (60)
A pet *n* = 2	The happiness, security and joy that comes with having a pet to care for	“My dog - welcoming and happy to see me” (37)
		“The dogs and the cat gives security” (30)
Hope for the future *n* = 2	To have a hope for the future. A belief that things will be sorted out e.g. faith in research	“Surf on the net regarding the disease - gives hope” (10)
Physical contact *n* = 3	Experience of physical contact, body touch and sex	“Physical contact, body contact” (33)
		“To have sex” (13)
Having a philosophy of life *n* = 1	A philosophy of life which gives comfort	“The Christian faith gives comfort” (29)
Being alone *n* = 1		“Be by myself.” (4)

^a^The number in the parenthesis represents the participant code

**Table 3:** Individual quality of life (IQOL): frequency of nominations among those with pain/without pain, level of satisfaction with IQOL among those with/without pain, level of pain severity and disease severity

Areas^a^	Frequency of nominations: participants with/without pain^b^ n (%)	Satisfaction with IQOL: participants with/without pain^c^, median (min-max)	Pain severity Index^d^ Mean (SD)	Bulbar function^e^, median (min-max)	Fine motor function^f^, median (min-max)	Gross motor function^g^, median (min-max)	Respiratory function^h^, median (min-max)
Social relations	35 (85)/12 (86)	6 (2-7)/6 (3-7)	2.8 (2.6)	10 (0-12)	8 (0-12)	7.5 (0-12)	12 (1-12)
Activities for amusement and relaxations	23 (56)/4 (28)	5 (1-7)/3.8 (1-7)	3.1 (2.6)	10 (0-12)	8 (0-11)	7 (0-12)	12 (1-12)
Being in the outdoor environment	12 (29)/7 (50)	5.5 (3-7)/5 (1-6)	1.6 (1.8)	10 (0-12)	8 (1-12)	8 (0-12)	12 (4-12)
Being independent	9 (22)/4 (28)	4 (1-6)/4.5 (1-6)	3.0 (2.8)	11 (3-12)	8 (1-11)	7 (2-12)	11 (4-12)
Being able to work	9 (22)/2 (14)	4 (1-6)/5.5 (5-6)	3.8 (2.8)	10 (4-12)	8 (1-12)	8 (5-12)	11 (4-12)
Being physically active	9 (22)/2 (14)	4 (1-7)/2 (1-3)	3.1 (2.9)	10 (3-12)	9 (1-12)	7 (0-12)	12 (5-12)
Acess to support and aids	8 (20)/2 (14)	6 (2-7)/6.5 (6-7)	3.6 (2.3)	10.5 (3-12)	7.5 (1-10)	5 (1-12)	12 (1-12)
Get along with daily inconveniences	6 (15)/3 (21)	3.5 (1-6)/3 (2-6)	2.7 (2.4)	8 (0-12)	7.5 (0-12)	7 (0-12)	10 (2-12)
Being in good health	3 (7)/4 (28)	6 (2-6)/5.5 (4-6)	2.3 (2.4)	9 (3-12)	8 (0-12)	5 (1-12)	11 (8-12)
A safe and comfortable home environment	6 (15)/ 0	6 (4-7)	4.2 (2.1)	10 (3-12)	6 (0-12)	8 (0-10)	12 (1-12)
The cottage	4 (10)/2 (14)	6 (5-7)/4 (1-7)	3.1 (2.8)	9.5 (3-12)	10 (0-12)	7.5 (2-10)	11.5 (5-12)
Enjoying good food and drinks	3 (7)/2 (14)	6 (4-6)/6.5 (6-7)	1.6 (1.6)	8 (3-12)	8 (1-11)	8 (0-12)	12 (4-12)
Being able to travel	4 (10)/1 (7)	6 (1-7)/5 (N/a)	4.3 (3.4)	12 (10-12)	10 (9-10)	8 (2-9)	12 (8-12)
Domestic care and family responsibilities	1 (2)/2 (14)	4 (N/a)/3.5 (3-4)	0.9 (1.6)	8 (7-10)	10 (5-12)	11 (5-12)	12 (10-12)
A pet	2 (5)/0	6.5 (6-7)	8.4 (0.9)	12 (11-12)	7 (4-7)	8 (7-9)	12 (11-12)
Hope for the future	2 (5)/0	3.5 (1-6)	2.6 (0.5)	11.5 (11-12)	7.5 (7-8)	3.5 (1-6)	8 (4-12)
Physical contact	1 (2)/2 (14)	4 (N/a)/1.5 (1-2)	2.4 (2.1)	8 (6-12)	3 (0-4)	4 (1-6)	11 (1-11)
Having a philosophy of life	1 (2) /0	5.5 (N/a)	1.3 (N/a)	8.0 (N/a)	6.0 (N/a)	8.0 (N/a)	12.0 (N/a)
Being alone	1 (2) /0	5 (N/a)	4.7 (N/a)	8.0 (N/a)	3.0 (N/a)	7.0 (N/a)	6.0 (N/a)

^a^ Areas nominated as important for individual quality of life (IQOL).  ^b^ participants with/without pain nominating the area. ^c^ How satisfied participants with pain were (1 – 7), where 1 indicate “as bad as could possibly be” and 7 “as good as could possibly be”. ^d^ How satisfied participants without pain were (1 – 7), where 1 indicate “as bad as could possibly be” and 7 “as good as could possibly be”. ^e^ Pain Severity Index (PSI) = average of ratings of worst, average and current pain during the past 24 hours. ^f^ Bulbar function, ^g^ fine motor function, ^h^ gross motor function and ^i^ respiratory function as subscales of the Amyotrophic Lateral Sclerosis Rating Scale - Revised version (ALSFRSR). Scores ranging from 0-12 on each subscale, where lower scores indicate worse function

### Individual quality of life and its association with pain, pain severity and disease severity

The median value for satisfaction with IQOL (index) for the total sample was 5 (25th percentile = 3.25 and 75th percentile = 6), representing a good IQOL according to the response format. There was no statistical difference in satisfaction with IQOL between participants with and without pain. Further, there was no statistically significant correlation between pain severity and satisfaction with IQOL, nor between the disease severity and satisfaction with IQOL for any of the four subscales representing disease severity. See Tables 3 and 4.

**Table 4:** Individual of quality of life and its association with pain severity (Brief Pain Inventory – Short form), disease severity (ALSFRS-R) and individual quality of life (SEIQOL-DW)

	**Pain severity** (*n* = 55)	**Bulbar function** (*n* = 61)	**Fine motor function** (*n* = 61)	**Gross motor function** (*n* = 61)	**Respiratory function** (*n* = 61)
**Individual quality of life**	r_s_^1^ = −.007	r_s_^1^ = .087	r_s_^1^ = .101	r_s_^1^ = .181	r_s_^1^ = .069
	*p* = .96	*p* = .50	*p* = .44	*p* = .163	*p* = .598

## Discussion

This study investigated whether pain prevalence and pain severity were associated with IQOL in patients with MND, which to the best of our knowledge not has been undertaken previously in the current population. Overall, satisfaction with IQOL was good and there was no difference in satisfaction with IQOL between participants reporting/not reporting pain. Correlations between pain severity and satisfaction with IQOL, and between disease severity and satisfaction with IQOL were weak and not statistically significant.

Seventy-four percent of the sample reported pain, and 71% had experienced pain during the past 24 h. Studies on pain prevalence in MND are still scarce and report that pain frequency varies between 15 to 84% [19]. Our figures were hence at the upper end of this range. Pain severity was on average rated to be moderate, rounded off to the lower end of the range. Fourteen percent reported severe pain, which differs from majority of studies reporting pain to be experienced as mild (< 3 on a 0-10 rating scale) with more severe pain in late stages of the disease [19]. Our study showed that moderate and severe pain could be present in samples with patients in non-terminal stages of the disease. It also confirmed figures reported by [11] pointing to that moderate to severe pain is not a consequence of one small sample only.

On a group level, satisfaction with current IQOL was good, which corresponds to previous studies using the SEIQOL-DW for such evaluation [29–31, 33]. There were some qualitative differences regarding what constitutes the most important areas for IQOL for those reporting pain and for those not reporting pain. Five areas were only expressed by those with pain: “*A safe and comfortable home environment”, “A pet”, “Hope for the future”, “Having a philosophy of life”,* and *“Being alone”.* The sample was too small to draw any conclusions of whether these qualitative differences between those reporting/not reporting pain are valid on a group level or just an expression of overall individual preferences.

Previous studies have reported that pain does not necessarily interfere with QOL in patients with ALS [8, 10], whereas Pizzimenti et al. [11] showed that this association lost its statistical significance when including depression as a covariate in the statistical analysis. We did not include any data on depression, but our results add to the hypothesis that pain in MND does not necessarily interferes with important aspects of quality of life. The weak associations between pain and IQoL could be an indication of that the participants experienced that pain was under control and possible to cope with. Good IQOL is predicted by coping strategies, in people with ALS [51], which strengthens this assumption. There are other aspects of pain that could be studied to gain further knowledge, e.g. certain pain types, frequency and unpredictability of pain flare-ups, which were perceived as stressful according to a recent study [52], and thus could have a negative impact on satisfaction with IQOL.

In line with previous studies, we found weak and statistically non-significant correlations between IQOL and disease severity [27, 29, 30, 32, 33]. Explanations might be similar to what is described regarding pain, i.e. that people with life threatening diseases tend to cope with their situation by accepting symptoms and disabilities in order to manage to continue life [53]. Additionally, the participants had relatively small impact from their disease on respiratory function, which is crucial for the patients’ survival [54]. Reduced variation in respiratory function may explain the absence of correlation between respiratory function as one indicator of disease severity and IQOL. Hence, further studies on respiratory deterioration and its impact on IQOL is recommended.

### Methodological considerations

There are some strengths and limitations of the study that deserve attention. Most importantly, we studied associations using a cross-sectional, and correlational design why causal inferences about the impact of pain on IQOL not could be drawn. Neither could we draw any conclusion about the influence of pain in relation to how IQOL varies/not varies over time. Our results thus add to the specification of hypotheses that need to be further investigated.

We used an established, standardized method to assess pain and pain severity, the BPI-SF, adhering to previous attempts to make pain studies in MND more comparable. Considering the construct of QOL, we chose an individual perspective on what areas of life that were important for a good quality of life when facing a degenerative disease. The individual perspective has high clinical relevance and can be a cornerstone when planning for the patient’s care. A limitation with the use of the measure could be if the participants may have chosen to nominate areas that were functioning well and omitted areas where they experienced physical limitations and negative emotions, resulting in a higher IQOL index [55].

The SEIQoL-DW offers an inductive method to collect and analyzing data, which is seldom strictly adhered to [38, 56]. In this study, participants were asked to nominate areas of importance all by themselves, without using the commonly applied prompt-list with pre-defined areas possible to nominate as important for QOL. This procedure secured the individual perspective and was well accepted and easily undertaken. To increase credibility of the inductive analysis, researcher triangulation was performed in several steps, and all accounts were sorted into areas after consensus agreements between researchers. To get a valid estimation of disease severity, we operationalized disease severity by reporting the results for the four subscales of ALSFRS-R separately, which has recently been recommended [50].

We recruited patients from four different MND teams to enhance variation and recruitment of as many participants as possible during a given time period. Patients with distinct cognitive impairment were not eligible for the study. Between 35 and 45% of the ALS population suffer from cognitive impairments [57], and our findings should therefore not be extrapolated to this subgroup of patients. We did not include patients with difficulties in speaking and understanding the Swedish language, and there was a limited number of participants who were in the severe stage of the disease, which both could threaten external validity. Finally, the proportion of participants with PLS was slightly higher compared to what is representative for the MND, that is expected to be 1–4% [5].

### Ethical considerations on risks and benefits of participation

Consideration was as far as possible given to the total time of the participants’ clinical visit, concerning the risk that participants were getting exhausted after their clinic visit due to the extra burden of study participation. The benefits of contributing to research by sharing experiences and reflections about their current life situation, and with the prospect to increase the understanding of what is important for people living with MND were assumed to exceed the burden of participation.

## Conclusions

The results add to the hypothesis that associations between pain prevalence and pain severity and IQOL are weak in MND. However, pain prevalence was high and the results pointed to that some participants experienced a high pain severity. Hence, pain can be a source of distress in itself, indicating that systematic tools for pain assessment and pain treatments tailored to the specific needs of the MND population should be developed and scientifically evaluated.

## Data Availability

The datasets used and/or analyzed during the current study are available from the corresponding author on reasonable request.

## Abbreviations

ALS: Amyotrophic lateral sclerosis
MND: Motor neuron disease
PLS: Primary Lateral Sclerosis
QOL: Quality of life
HRQOL: Health related quality of life
IQOL: Individual quality of life
Modified Swedish SEIQoL-DW: The Modified Swedish Schedule for the Evaluation of Individual Quality of Life - Direct Version
ALSFRS-R: The Amyotrophic Lateral Sclerosis Functional Rating Scale - Revised Version
BPI-SF: The Brief Pain Inventory – Short-form
